# Oxidative Stress and Lipid Mediators Modulate Immune Cell Functions in Autoimmune Diseases

**DOI:** 10.3390/ijms22020723

**Published:** 2021-01-13

**Authors:** Piotr Wójcik, Agnieszka Gęgotek, Neven Žarković, Elżbieta Skrzydlewska

**Affiliations:** 1 Department of Analytical Chemistry, Medical University of Bialystok, 15-222 Bialystok, Poland; piotr.wojcik@umb.edu.pl (P.W.); agnieszka.gegotek@umb.edu.pl (A.G.); 2 Laboratory for Oxidative Stress, Rudjer Boskovic Institute, 10000 Zagreb, Croatia; Neven.Zarkovic@irb.hr

**Keywords:** lipid mediators, endocannabinoids, ROS, prostaglandins, oxidative stress, immunity, rheumatoid arthritis, psoriasis, psoriatic arthritis, systemic lupus erythematosus

## Abstract

Autoimmune diseases, including psoriasis, systemic lupus erythematosus (SLE), and rheumatic arthritis (RA), are caused by a combination of environmental and genetic factors that lead to overactivation of immune cells and chronic inflammation. Since oxidative stress is a common feature of these diseases, which activates leukocytes to intensify inflammation, antioxidants could reduce the severity of these diseases. In addition to activating leukocytes, oxidative stress increases the production of lipid mediators, notably of endocannabinoids and eicosanoids, which are products of enzymatic lipid metabolism that act through specific receptors. Because the anti-inflammatory CB2 receptors are the predominant cannabinoid receptors in leukocytes, endocannabinoids are believed to act as anti-inflammatory factors that regulate compensatory mechanisms in autoimmune diseases. While administration of eicosanoids in vitro leads to the differentiation of lymphocytes into T helper 2 (Th2) cells, eicosanoids are also necessary for the different0iation of Th1 and Th17 cells. Therefore, their antagonists and/or the genetic deletion of their receptors abolish inflammation in animal models of psoriasis—RA and SLE. On the other hand, products of non-enzymatic lipid peroxidation, especially acrolein and 4-hydroxynonenal-protein adducts, mostly generated by an oxidative burst of granulocytes, may enhance inflammation and even acting as autoantigens and extracellular signaling molecules in the vicious circle of autoimmune diseases.

## 1. Literature Review

Autoimmune diseases are a group of diseases in which the immune system becomes activated against host cells. These diseases are serious social and medical problems affecting many people, reducing their quality of life and even lifespan. Nevertheless, despite the enormous effort put into developing an effective therapy, even the latest so-called biological therapies, which are much more effective and safer than the immunosuppressive drugs used so far, are still not able to cure but only temporarily reduce disease symptoms. Also, because these therapies modulate the immune system, they cause side effects such as susceptibility to infections [1]. This therapeutic problem may be because autoimmune diseases and their underlying pathophysiology are not fully understood. Nevertheless, it is now known that autoimmune diseases are the result of a combination of genetic and environmental factors, the latter of which are very often considered to act as “triggers” because they are not the main cause of the disease but rather help their development in already genetically susceptible individuals. This is true for the most common autoimmune diseases such as psoriasis, systemic lupus erythematosus (SLE), and rheumatoid arthritis (RA) [2,3,4].

Psoriasis, which affects about 3% of the population in western countries [5], has different clinical subtypes; the most common types are psoriasis vulgaris and psoriatic arthritis. Clinically, psoriasis is manifested by characteristic skin lesions, and in the case of psoriatic arthritis, is accompanied by pain and malfunctions of joints. Psoriasis is assumed to be a multifactorial disease, which develops in individuals that have a genetic predisposition for the onset of the disease and is further enhanced by environmental factors. This is supported by the fact that psoriasis is up to 35% more common in twins than in other people [6], being inherited in a multi-genetic way with over 40 alleles associated with psoriasis. Clinical symptoms arise from the activity of immune cells (leukocytes), while changes of leukocyte phenotype and biochemical features have been observed in not only the skin but also the blood of patients with psoriasis vulgaris [5]. 

Similar to psoriatic arthritis, RA is a disease whose symptoms affect joints, while some changes can also be seen in blood cells, allowing RA to be considered a systemic disease, too [4]. Synovial hyperplasia is a hallmark of the disease manifested by excessive proliferation of fibroblast-like synovial cells in the joints (driven by inflammatory cytokines), which reduces the mobility of the joints. The disease is, like other autoimmune diseases, caused by a combination of environmental and genetic factors. However, despite some similarities of symptoms, psoriatic arthritis and RA have a significantly different pathogenesis. Importantly, bacterial infections or smoking are the most important triggers for RA symptoms because these factors can lead to a pathological response of the immune system. The disease affects up to 1% of the population, particularly targeting the elderly [7]. 

Another autoimmune disease associated with chronic inflammation with complex, but not fully understood pathogenesis, is SLE. Although less common than psoriasis or RA, with 20–200 cases per 100,000 individuals, the course of the disease is more severe. SLE has complex symptoms that affect different tissues, including painful and swollen joints, fever, chest pain, hair loss, mouth ulcers, swollen lymph nodes, tiredness, and red rash showing systemic characteristics of the disease [8].

**Figure 1:**
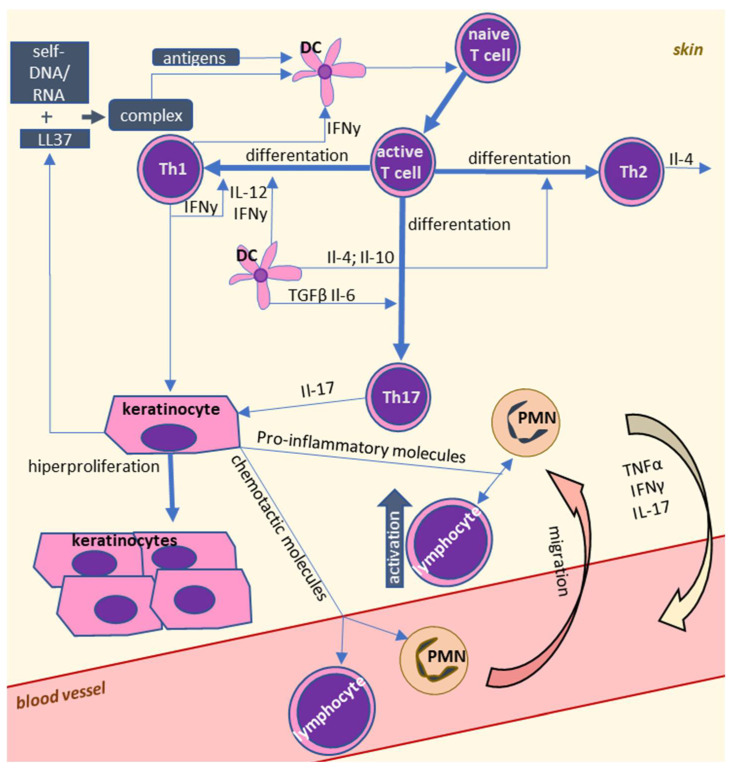
Cellular interactions in psoriasis. Psoriasis is characterized by abnormal interactions between leukocytes. Overactivation of dendritic cells leads to polarization of T lymphocytes into T helper 1 (Th1) and in consequence higher levels of pro-inflammatory factors produced by these cells, which cause proliferation of keratinocytes. Activation of granulocytes during psoriasis is important for the progression of inflammation and oxidative stress.

**Figure 2:**
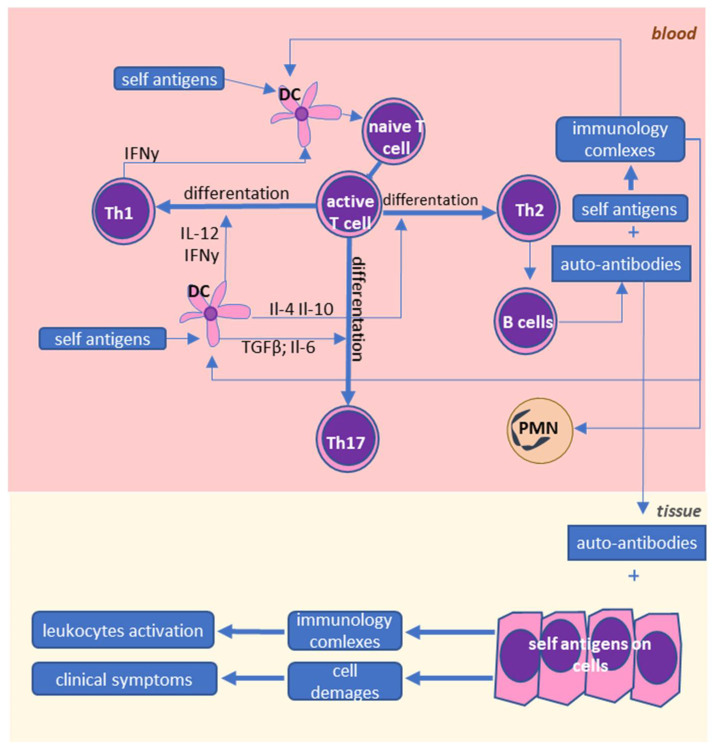
Cellular interactions in lupus erythematosus (SLE) and rheumatoid arthritis (RA). SLE and RA are characterized by abnormal interactions between leukocytes. Overactivation of dendritic cells leads to higher activation of different subpopulations of T lymphocytes, and in consequence, to higher levels of pro-inflammatory molecules produced by these cells. Moreover, Th2 activates B cells, which leads to the production of autoantibodies that bind to the self-antigens, in consequence leading to the destruction of tissues and further activation of the immune system.

The most important cells for the development of autoimmune diseases seem to be lymphocytes, particularly T cells in both forms of psoriasis (Figure 1), and T and B cells in RA and SLE (Figure 2). In healthy individuals, T cells are responsible for the development of adaptive immunity and modulation of the immune system. The T cells that have not been previously activated are denoted as naive lymphocytes, which are activated by monocytes and dendritic cells in a process of antigen presentation. Dendritic cells can only activate naive lymphocytes, while monocytes are able to activate also memory lymphocytes [9]. Usually, dendritic cells recognize pathogens by Toll-like receptors (TLRs) before they phagocytose them. Pathogens are proteolyzed and their fragments (i.e., antigens) are complexed by major histocompatibility complex II (MHC II) molecules and transported to the cell surface where they are presented. Antigens presented by MHC II are recognized by T-cell receptors (TCRs) and cluster of differentiation 4 (CD4) receptors on Th lymphocytes. Moreover, costimulatory molecules such as CD80 or CD86 are also present on dendritic cells [10]. The presence of both MHC II-presented antigen and costimulatory molecules is necessary for the activation of Th lymphocytes. TCRs are characterized by a high degree of diversity, and only lymphocytes that express TCRs specific to particular antigens are activated upon their encounter.

It has been suggested that the selectivity of Toll Like Receptors (TLRs) is disturbed in psoriasis where dendritic cells are activated despite the absence of pathogens to be eliminated. Two specific TLRs, TLR-9 and TLR-7, are activated by DNA and RNA, respectively [11]. However, they are activated by “foreign” nucleic acids and not by the own human nucleic acids. Human DNA is normally digested by deoxyribonucleases (DNases) and a lack of DNase 1 is an important factor for the onset of autoimmune diseases [12]. Moreover, TLR-7 and TLR-9 are endosomal receptors so they can only be activated by DNA or RNA that has been taken up by cells through endocytosis. Moreover, TLR-7 can only be activated by double-stranded RNA, which is typical for viruses, not for mammalian genomic material [13]. In the case of psoriasis, large amounts of the antimicrobial peptide LL-37 are produced (mainly by keratinocytes). This peptide forms complexes with nucleic acids, which prevents nucleic acids from being digested, but such complexes undergo endocytosis, activating TLRs and, consequently, dendritic cells and T lymphocytes.

TLRs are also important for the development of SLE. In SLE, high TLR7 expression correlates with disease severity. Moreover, some polymorphisms in the TLR-7 gene correspond to a higher risk of SLE [14,15]. Additionally, administration of a TLR-7 agonist—imiquimod—leads to systemic changes in animal models [16]. As activation of dendritic cells leads to activation of lymphocytes, these changes may cause the pro-inflammatory phenotype of lymphocytes. In RA, high expression of TLRs has been observed in synovial fibroblasts and monocytes [17]. Moreover, RNA from the synovial fluid of RA patients acts as a ligand for TLRs and leads to monocytic activation, whereas RNA from the plasma of these patients, or healthy subjects, does not [18]. Even though there is no direct evidence that RA is associated with increased expression of TLRs in dendritic cells, the presence of TLR ligands (including RNA) in the synovial fluid suggests that immune cells present in joints are activated through TLRs. Moreover, in the case of osteoarthritis, dendritic cells show higher TLR expression [19].

Autoimmune diseases are typically accompanied by a Th1/Th2 imbalance in favor of Th1, with more recent data showing that Th17 cell numbers can also increase. Th1 and Th17 cells play a role in immunity and in the development of autoimmune diseases through the production of large amounts of cytokines IFNγ and IL-17, respectively. IFNγ, the level of which correlates with disease activity [20], stimulates monocyte activation, antigen presentation, and differentiation of lymphocytes to Th1 cells, thereby enhancing immune responses and inflammation [21,22]. In psoriasis vulgaris, IFNγ is also responsible for the excessive proliferation of keratinocytes. It has been found that adding Th cell-conditioned medium to keratinocytes causes their excessive proliferation, while IFNγ inactivation abolishes this effect [23]. Moreover, B cells are also important in the development of SLE and RA because they produce autoantibodies. In contrast to Th cells, which are mainly regulatory cells, the main biological role of B lymphocytes is the production of antibodies. Activation of B cells begins when an antigen binds to a B-cell receptor (BCR). After binding, the antigen is degraded into peptide fragments in the cell and the fragments are presented on the cell surface. The antigens are then recognized by specific, previously activated Th cells. The interaction between the CD40 molecule on the surface of B lymphocytes and CD40L on T lymphocytes is necessary for B cell activation. Additionally, Th cells produce cytokines that play an important role in B cell activation [24]. After activation, B cells develop into plasma cells and produce immunoglobulin (IgM and IgG), and/or B memory cells. B cells are only activated by Th cells when both cells react to the same antigen.

In SLE, B cells produce several autoantibodies, including antibodies against double-stranded DNA (anti-dsDNA) and anti-nuclear antibodies (ANAs) [25]. These antibodies appear to be important in the pathogenesis of autoimmune diseases. When antibodies bind to their target, they form immune complexes, which are potent activators of immune cells. Moreover, levels of autoantibodies correlate with the severity of the disease and are the most important diagnostic markers for SLE. In RA, the most important antibodies are against the fragment crystallizable region (Fc region) of IgG, which is also called rheumatoid factor (RF) [26]. Diagnosis of RA is highly dependent on the presence of RF in serum. As RA has symptoms resembling other diseases (including psoriatic arthritis), its diagnosis is confirmed only if the patient is RF positive. Although autoantibodies (anti-dsDNA and ANA) may also be present in psoriatic patients, they are observed only in the minority of patients and are thus not a significant clinical feature of the disease [27]. 

Neutrophils also play a significant role in the development of autoimmune diseases. They are the most numerous cells among leukocytes—constituting over 50% of peripheral blood leukocytes—and are the first to react at the site of inflammation. The presence of microbes or tissue damage leads to the expression of P-selectin, E-selectin, ICAM-1, and VCAM-1 on endothelial cells. As neutrophils typically circulate in the bloodstream near blood vessel walls, they recognize and interact with these endothelial membrane molecules, which leads to their activation and migration through vessel walls in a process called diapedesis [28]. Diapedesis is additionally intensified by chemoattractants, mainly of bacterial origin, like N-formylmethionyl-leucyl-phenylalanine (fMLP) [29]. Moreover, once in tissue, neutrophils move toward microbes thanks to their ability to chemotaxis, that is, to move under the influence of chemical signals, and consequently move toward a higher concentration of the above-mentioned chemoattractants. Endogenous lipid mediators produced during inflammation, such as leukotrienes B4 (LTB4), also act as chemoattractants for neutrophils [30], increasing their recruitment to the site of inflammation.

Neutrophils also have pattern recognition receptors (PPRs), which react with pathogen-associated molecular patterns (PAMP), which are molecules not produced by human cells but by microbes, as in the case of bacterial lipopolysaccharide (LPS) and viral double-stranded RNA [31]. The main function of neutrophils is phagocytosis and elimination of pathogens. When a pathogen is phagocytosed, it is eliminated by neutrophils through oxygen-dependent or -independent mechanisms. In oxygen-dependent mechanisms, neutrophils produce large amounts of reactive oxygen species (ROS) to eliminate pathogens, whereas in oxygen-independent mechanisms, antimicrobial proteins in the granules of neutrophils digest the phagocytosed bacteria. In addition, antimicrobial substances stored in neutrophils can also be released outside in a process called degranulation, or they can be mixed with chromatin inside neutrophils and released as a network of chromatin and antimicrobial peptides in a cell death process called NETosis [32].

### 1.1. Oxidative Stress

Autoimmune diseases are usually accompanied by metabolic changes that involve the development of pro-inflammatory processes and oxidative stress. In psoriasis, redox imbalance is observed not only in skin cells but also in plasma and blood cells, including granulocytes and lymphocytes [33,34]. Moreover, it has also been shown that mutations in genes encoding some antioxidants, such as paraoxonase-1, correlate with the severity of the disease [35]. As a result, higher levels of oxidatively modified lipids and proteins can also be observed [33,34,36]. Therefore, it has been suggested that a diet rich in antioxidants should treat psoriasis. It has been found that the antioxidant astilbin reduces both the production of ROS and the proliferation of keratinocytes, suggesting the important role of ROS-induced changes in immune cells and keratinocytes [37].

Similar to psoriasis, oxidative stress is also involved in the development of SLE and RA and can be an important factor in the onset of these diseases [38,39]. In SLE, oxidative stress causes DNA damage, which leads to the production of anti-dsDNA antibodies [40]. Hence, therapy with the antioxidant-glutathione precursor N-acetylcysteine, or with other antioxidants such as metformin, has a positive impact on the health condition of patients with SLE [41,42]. Opposite to that, smoking, which also leads to oxidative stress, is the most important risk factor in RA [43,44,45]. Oxidative stress may not only trigger RA but is also involved in its pathogenesis. Namely, neutrophils are known to generate large amounts of ROS and are the most abundant cells in the synovial fluid of RA patients where they generate higher levels of total ROS and hydroxyl radicals—the levels of which correlate with disease activity score (DAS28), which is the most common clinical factor used to determine the severity of symptoms of RA [46]. Neutrophils are also the main source of pro-oxidative molecules such as myeloperoxidase (MPO), the levels of which correlate with levels of the pro-inflammatory cytokines IL-8 and IL-18 in RA. Standard treatment for RA leads to a decrease in levels of MPO and these cytokines, supporting the observation that inflammation and oxidative stress occur together during RA [47]. 

Since neutrophils generate a large amount of ROS, their activation is important for the onset of oxidative stress, which is defined as a shift in the redox balance toward oxidative reactions. As a result, ROS interact with proteins, nucleic acids, and especially lipids, causing oxidative modifications of their structure. One of the metabolically significant consequences is the peroxidation of membrane phospholipids with increased production of reactive aldehydes such as 4-HNE [48]. This leads to serious metabolic disturbances and can even lead to cell death [49]. ROS also lead to the activation of transcription factors that modulate the biosynthesis of antioxidant proteins and pro-inflammatory factors [33]. Therefore, oxidative-stress-dependent activation of transcription factors can exacerbate inflammation and activate immune cells [33,34]. 

ROS activate transcription factors such as NF-κβ and Nrf2. The NF-κβ is activated by over 150 other factors, including cytokines (e.g., TNF-α, TGF-α, IL-1, IL-17), TLR receptors, DNA damaging factors (including oxidative stress), hypoxia, H_2_O_2_, and oxidized low density lipoproteins (LDL). In turn, NF-κβ activates the transcription of factors such as IFN-γ, IL-1, TNF-α, TNF-β, p62, p53, etc. [50,51]. Psoriasis, both vulgaris and arthritic, as well as SLE and RA, have higher expression of NF-κβ and TNF-α, which is the product of its activity [33,34,52]. In contrast, inhibition of TNF-α action leads to improvement of the clinical condition of patients suffering from these diseases. Therefore TNF-α antagonists (adalimumab, infliximab, and etanercept) were approved by the US Federal Drug and Administration (FDA) for the treatment of psoriasis and rheumatic arthritis [53,54,55,56]. 

Similarly, Nuclear factor erythroid 2-like(Nrf2) expression is also enhanced in these diseases. However, the Nrf2 transcription factor, which binds to antioxidant response elements in DNA, enhances the expression of proteins responsible for defense against oxidative stress [57]. Its activation is mainly caused by ROS, which may inactivate the Nrf2 cytosol inhibitor Keap1. Under normal, unstressed conditions, Nrf2 is present in cells in a complex with Keap1, determined for proteasomal degradation. However, under conditions of oxidative stress, Keap1 is oxidized, resulting in the release of Nrf2, which can then be translocated to the nucleus. It has been observed that Nrf2 activation in psoriasis leads to higher expression of keratins K6, K16, and K17, which enhance the proliferation of keratinocytes [58]. In Nrf2^−/−^ mice, higher DNA oxidation and production of antibodies against DNA have been observed. Moreover, higher levels of cytokines and clinical symptoms similar to SLE have also been observed, which suggests that dysregulation of the Nrf2 system may be important in the development of SLE [59]. In addition, in a mouse model of pristane-induced lupus nephritis, administration of the Nrf2 activator dimethyl fumarate was found to cause alleviation of inflammation, providing additional evidence that Nrf2 activation has an anti-inflammatory role in autoimmune diseases similar to SLE [60]. Nrf2 also seems to be able to modulate cellular function in RA because the administration of S-propargyl-cysteine, which is a potent activator of the Nrf2 pathway, leads to alleviation of RA symptoms in a rat model and reduces the production of inflammatory cytokines in human rheumatoid fibroblast-like synoviocytes [61].

Oxidative-stress-induced modifications to transcription factors and intracellular signaling pathways may strongly affect functions of leukocytes, particularly dendritic cells. In vitro studies have shown that oxidative stress leads to activation of dendritic cells [62], the production of IL-8 and TNF-α by these cells, and enhanced TLR expression, which may play an important role in their abnormal response to autoantigens [63]. It has been shown that in the presence of vitamins C and E, the activation of dendritic cells is weaker and that lower amounts of cytokines, such as IL-1β, IL-12, TNF-α, and IFN-γ, are formed in vitro after administration of pro-inflammatory factors [64]. Moreover, lymphocytes co-cultured with dendritic cells previously treated with these vitamins are characterized by reduced proliferation and a shifted response toward Th2, i.e., production of IL-10, IL-4, and IL-5. In the presence of these antioxidant vitamins, the phosphorylation of the p38 mitogen activated protein kinase (MAPK) kinase also decreases, indicating inhibition of the p38 MAPK pathway [64]. This pathway is activated, among others, by UV, oxidative stress, and pro-inflammatory cytokines and leads to overproduction of TNF-α and IL-1, as well as apoptosis [65,66]. A decrease in ROS levels in dendritic cells was also confirmed after incubation with these antioxidant vitamins [64]. 

Vitamins are not the only antioxidants that have been studied for their effects on dendritic cells. Bursopentine is an antioxidant that has been shown to inhibit the production of nitric oxide (NO) in LPS-activated dendritic cells, which is accompanied by suppression of LPS activity, observed as a decrease in the production of TNF-α as well as inhibition of the maturation of these cells [67]. On the other hand, increasing ROS levels leads to increased activation of lymphocytes by dendritic cells [68]. These metabolic modifications are accompanied by the enhancement of lipid peroxidation in dendritic cells. Since activation of dendritic cells and interactions between dendritic cells and lymphocytes appear to be crucial for the development of autoimmune diseases, oxidative stress may be associated with the development of autoimmune diseases through action on dendritic cells [67]. Oxidative stress induces T lymphocyte polarization by enhancing their interaction with dendritic cells and may also directly increase Th17 development via the mammalian target of rapamycin (mTOR) pathway [69,70]. Antioxidants like resveratrol, which can inhibit the mTOR pathway, are known to have a positive effect in murine models of immunologic diseases [71]. In the case of B lymphocytes [72], their increased activation may be caused by increased activity of T lymphocytes, higher cytokine levels, or the direct influence of ROS. Oxidative stress caused by hydrogen peroxide has been shown to result in higher activation and maturation of B lymphocytes and may accordingly also contribute to the development of diseases in which malfunction of B cells leads to the production of autoantibodies.

**Figure 3:**
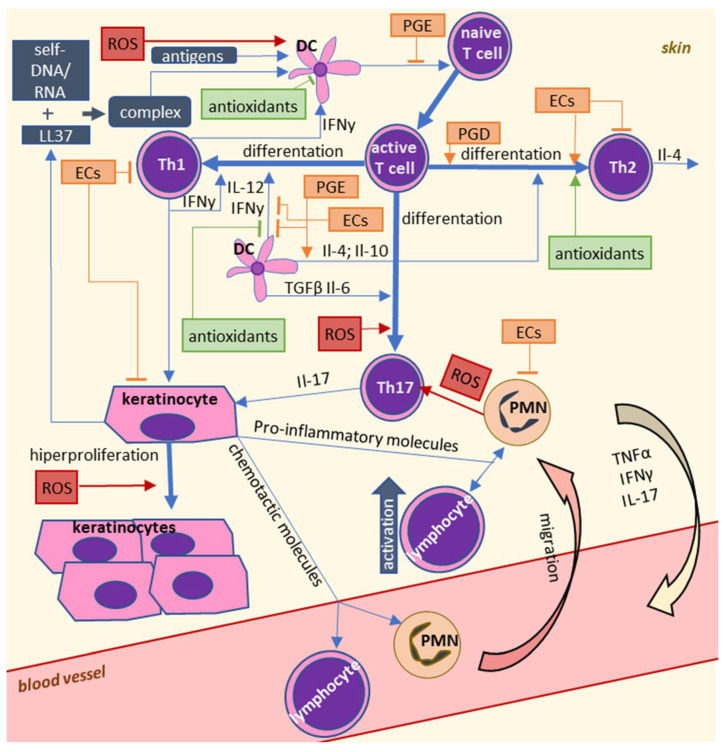
Influence of reactive oxygen species (ROS) and lipid mediators on immune cell interactions in psoriasis.

**Figure 4:**
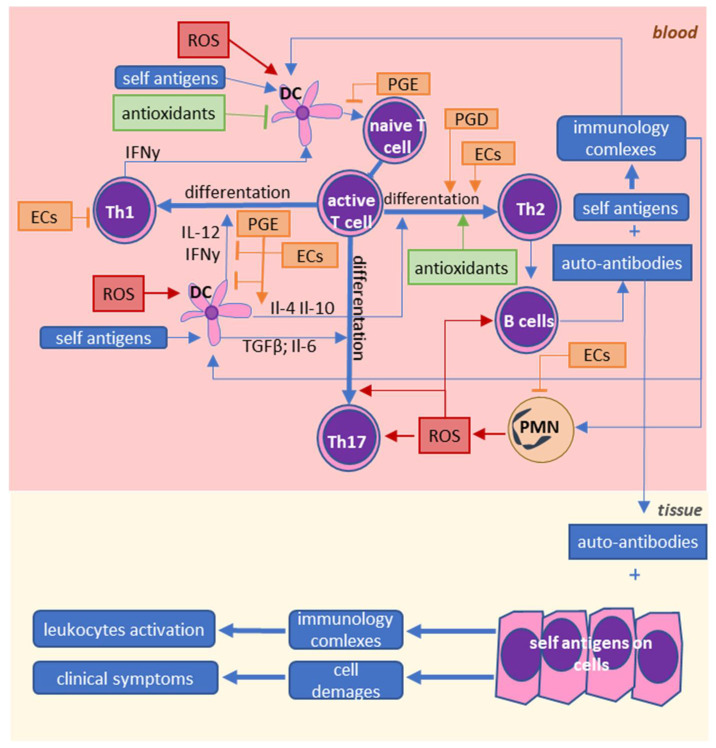
Influence of reactive oxygen species and lipid mediators on immune cell interactions in SLE and RA.

Taken together, these findings suggest that oxidative stress may be an important factor for the development of autoimmune diseases. It is well known that oxidative stress is caused by inflammation, but induced oxidative stress may also enhance inflammation. However, it is not certain whether oxidative stress is only a consequence, or it may also be the primary cause of inflammation in autoimmune diseases. Even if oxidative stress is a result of inflammation, it causes a further increase in the activity of leukocytes. Moreover, oxidative stress leads to significant changes in lipid metabolism, and lipid metabolites may also be involved in the pathophysiology of autoimmune diseases as shown by Figure 3 and Figure 4. 

In addition to some lipid mediators, ROS affect the pathophysiology of psoriasis by interacting with leukocytes at the very beginning of the inflammatory process. They may thus be triggers for the development of the disease, but they may also intensify the proliferation of keratinocytes, thereby intensifying symptoms of psoriasis. On the other hand, some lipid mediators, particularly endocannabinoids, seem to be anti-inflammatory factors.

ROS and lipid mediators play important roles in the onset of the pathological interactions between different leukocytes in SLE and RA. First, they are involved in regulating the activity of dendritic cells and the differentiation of lymphocytes. Afterward, they further activate other leukocytes, thereby enhancing inflammation.

### 1.2. Lipid Mediators

**Figure 5:**
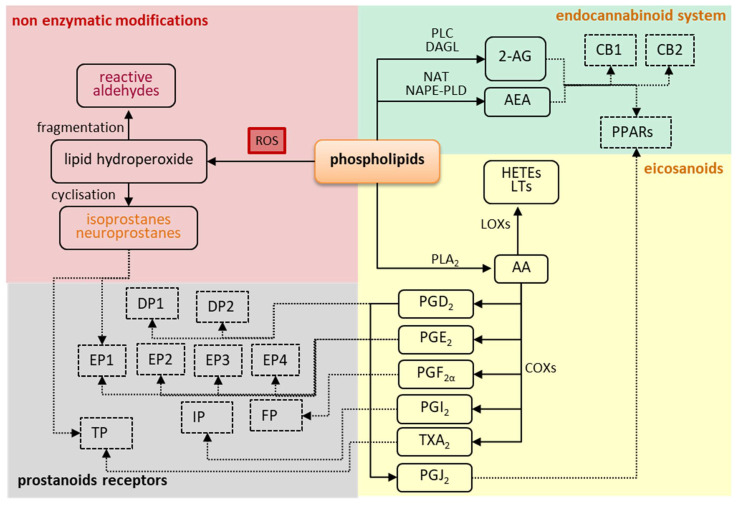
The most important lipid derivatives are generated from arachidonic acid in enzyme-dependent pathways and their receptors. Abnormal lipid metabolism is observed during oxidative stress. Non-enzymatic modifications involve the fragmentation and cyclization of lipids, leading to the formation of reactive aldehydes and isoprostanes, respectively. On the other hand, endocannabinoids and prostanoids are generated through the enzymatic pathway. These compounds affect cell functions by activating different receptors. Importantly, one compound may act on different receptors, while one receptor can be activated by different compounds.

It is well known that oxidative stress promotes modification of lipid metabolism [34,73,74]. Oxidative conditions have been shown to promote the activation of enzymes such as phospholipase, cyclooxygenases (COX), lipoxygenases (LOX), and cytochrome p450 (CYP450) [75], which are involved in the metabolism of lipids and their derivatives, resulting in the formation of eicosanoids, which are, in turn, involved in modulation of the redox balance and inflammation by activating specific receptors. Phospholipids are also metabolized by N-acyltransferase (NAT), phospholipase C (PLC), diacylglycerol lipase (DAGL), and N-acylphosphatidylethanolamine phospholipase D (NAPE-PLD) into endocannabinoids (Figure 5) [76,77]. Moreover, oxidative conditions enhance ROS-dependent lipid metabolism, resulting in an increase of both oxidative fragmentation and oxidative cyclization of lipid hydrocarbon chains. The oxidative stress observed in autoimmune diseases leads to elevated levels of various lipid mediators. Genetic studies have confirmed that in at least some autoimmune diseases, lipid mediators are not only the result of oxidative stress and inflammation but also play an important role in modulating these processes [78]. 

#### 1.2.1. Endocannabinoids

Endocannabinoids are a large group of ester, ether, and amide derivatives of fatty acids, of which the best-known mediators of cellular metabolism are anandamide (AEA) and 2-arachidonoyl glycerol (2-AG) [76,77]. Endocannabinoids are mainly biosynthesized from phospholipids present in the cell membrane. AEA synthesis begins when arachidonic acid is transferred from phosphatidylcholine to phosphatidylethanolamine, so thus-formed N-arachidonoyl phosphatidylethanolamine is then hydrolyzed to AEA by phospholipase A_2_, C, or D [79]. However, the synthesis of 2-AG is catalyzed by diacylglycerol lipase, which hydrolyzes phosphatidylinositol [80]. Due to biological properties similar to endocannabinoids (activation of the same receptors), phytocannabinoids were discovered, of which cannabidiol and tetrahydrocannabinol are the best known for their effects on cellular metabolism [81,82,83].

Apart from other functions that include regulation of leukocyte metabolism, endocannabinoids fulfill their metabolic role in the body mainly through the activation of G protein-coupled receptors. Among them, the most important are the cannabinoid receptors (CB1 and CB2), which have opposing effects relative to each other [76,77]. Activation of CB1 has pro-oxidative and pro-inflammatory effects, while CB2 activation enhances antioxidant and anti-inflammatory conditions [76,77]. Because CB2 is abundant in immune cells, endocannabinoids are considered to be the main regulators of inflammation, so the increased activation of cannabinoid receptors in autoimmune diseases is very often viewed as a protective mechanism [84]. This is supported by the fact that mutations in the CB2 receptor may lead to higher lymphocyte activity. Various mutations, including the nonsense mutations in enzymes that synthesize endocannabinoids, correlate with a higher risk of some autoimmune diseases [85,86]. Moreover, other receptors like peroxisome-proliferator-activated receptors (PPARs), especially PPAR-δ and PPAR-γ, are activated by endocannabinoids and act in an immunosuppressive manner [87]. 

On the other hand, phytocannabinoids, particularly cannabidiol (CBD), are able to inhibit neutrophil migration and ROS production, as CBD has been shown to cause a significant decrease of ROS levels in neutrophils in vitro [88]. The 2-AG also reduces neutrophil migration and degranulation, as well as ROS generation in the cells [89,90]. It may therefore be suggested that, by inhibiting the main mechanisms of action of neutrophils, cannabinoids can be important negative regulators of neutrophils and innate immunity.

Endocannabinoids also inhibit the synthesis of pro-inflammatory cytokines (e.g., IL-6, IL-12, and IFNγ) by dendritic cells that can thus impact the differentiation of lymphocytes to Th2 and prevent their differentiation to Th1 [91]. Endocannabinoids may also cause apoptosis of dendritic cells, thereby preventing the activation of lymphocytes and the development of adaptive immunity [92]. Moreover, it has been shown that endocannabinoids inhibit both activated Th1 and Th2 lymphocytes, so their addition to the cells in vitro decreases their production of cytokines (TNF-α, IL-6, and IL-8, IFNγ) [91]. On the other hand, it is also suggested that endocannabinoids cause a shift of the lymphocytic profile to Th2 by inducing the production of IL-4 and IL-10 [93]. Therefore, endocannabinoids may be involved in T lymphocyte function, directly changing their phenotype and indirectly by modulating dendritic cell functions, thus changing the environment in which lymphocytes act and leading to lower cellular activation and changing their profile to Th2. 

As with most autoimmune diseases, in psoriasis, SLE, and RA, the lymphocyte profile is shifted to Th1, and endocannabinoids may play an important role as negative regulators of autoimmune diseases. This may underpin certain compensatory mechanisms, especially in cases where endocannabinoid levels are elevated, as is the case in autoimmune diseases. In fact, in psoriasis vulgaris, levels of AEA and 2-AG, as well as expression of CB2 receptors, are enhanced in granulocytes and peripheral blood mononuclear cells (PBMC), while in psoriatic arthritis, expression of CB2 receptors is decreased in spite of elevation of endocannabinoids [33,34]. Therefore, exacerbation of psoriasis vulgaris to psoriatic arthritis may be caused by disturbances in the endocannabinoid system. However, in vitro studies have shown that CBD reduces inflammation and oxidative stress in keratinocytes in both healthy individuals and patients with psoriasis vulgaris, which may indicate beneficial properties of CBD [94]. Moreover, CBD also reduces the formation of a NETotic network in neutrophils [95]. However, topical application of CBD-containing oil decreases the Psoriasis Area and Severity Index (PASI) score in patients with psoriasis vulgaris [96]. However, in the case of SLE, plasma levels of 2-AG are also increased, which may result in decreased activity of lymphocytes. Similarly, higher levels of endocannabinoids have been observed in the synovial fluid of RA patients [97]. In addition, there is direct evidence that cannabinoids act in an immunosuppressive manner in RA [98,99] and that CBD, similar to the synthetic selective CB2 agonist O3223, reduces the severity of symptoms in animal models of the disease [98,99]. 

Therefore, the widely recognized anti-inflammatory effects of endocannabinoids appear to be well documented in autoimmune diseases. The action of these mediators alone will likely not be enough to reduce inflammation, since many other pro-inflammatory factors are also relevant for autoimmune diseases. Nevertheless, a positive response to cannabinoid-based treatment clearly indicates that the endocannabinoid system might dampen the activity of the immune system in autoimmune diseases [96,98]. 

#### 1.2.2. Eicosanoids

Eicosanoids are another group of lipid mediators formed as a result of enzymatic metabolism of polyunsaturated fatty acids. The enzyme catalyzing the formation of eicosanoids is cyclooxygenase (COX-1 and COX-2). The COX-1 isoform is constitutively expressed, while COX-2 expression is highly dependent on the cellular environment [100]. However, inflammation or pro-inflammatory factors lead to overexpression of COX-1 [101].

The main substrate of cyclooxygenases is arachidonic acid (AA), which is released from phospholipids by phospholipase A_2_ [75]. Due to COX activity, AA is metabolized into two-series eicosanoids, initially resulting in the formation of prostaglandin G_2_ (PGG_2_), which is subsequently reduced to PGH_2_, after which it is rapidly converted to other prostaglandins (e.g., PGE_2_, PGF_2_, PGD_2_, PGI_2_) and thromboxanes (e.g., thromboxane A_2_) via specific prostaglandin and thromboxane synthases [102]. Cyclooxygenases also metabolize other substrates such as endocannabinoids into AA, which may also be metabolized via the LOX and cytochrome P450 pathways with, e.g., hydroxyeicosatetraenoic acid (HETE) generation [102].

Prostaglandins are generally pro-inflammatory compounds, but their effect on immune cells is complex and is at least partially dependent on the activation of their receptors. Prostaglandins act through specific prostanoid receptors—namely prostanoid E receptors (EP1, EP2, EP3, and EP4) for PGE_2_; prostanoid D receptors (DP1 and DP2) for PGD_2_; prostanoid F receptors for PGF_2α_; prostanoid I receptor for PGI_2_ and the thromboxane receptor for TXA_2_ [103,104,105,106]. Although no specific receptors for PGJ_2_ have been characterized so far, PGJ_2_ is an agonist of both the DP1 and DP2 receptors [103,107]. Prostaglandins may also activate PPAR receptors [107], and the diversity of receptors creates options for different cellular responses to prostaglandins, depending on the dominant activation of the receptor [103,104].

Prostaglandins play important role in regulating the differentiation of lymphocytes into different cellular subpopulations. In the case of dendritic cells, the action of PGE_2_ limits the ability of these cells to activate naïve T lymphocytes. The TXA_2_ appears to suppress the interaction between dendritic and Th cells and thus reduces Th cell differentiation both in vitro and in vivo. Moreover, in the presence of PGE_2_, dendritic cells produce lower amounts of IL-12 and higher levels of IL-10, which leads to the differentiation of T cells into Th_2_ cells [108,109]. PGD_2_ also promotes the differentiation of T lymphocytes into Th_2_ cells and thus may indirectly promote B cell activation [110,111]. These data indicate the involvement of prostaglandins in the immune response and the generation of the Th2 phenotype. On the other hand, EP1^−/−^ mice show a diminished Th1 response, suggesting that PGE_2_ is also necessary for the development of a Th1 response [112]. Moreover, EP1 agonists in vitro promote Th1 responses [112], while PGE_2_ in vivo induces Th17 cell differentiation and the production of pro-inflammatory cytokines [113]. In contrast, aspirin, which is a COX-2 inhibitor, inhibits the development of Th1 and Th2 lymphocytes [114]. Moreover, in vitro COX-2 inhibition reduces B cell maturation and antibody production, for which EP2 and EP4 receptors are the most important as their agonists affect B cell activation [115,116].

Both forms of psoriasis are characterized by increased activity of phospholipase A_2_ and COX-2 in lymphocytes, which favors increased levels of eicosanoids, particularly of the prostaglandin PGE_2_ [34]. The interactions of Th lymphocytes, their activities, and the balance between their subpopulations appear to be crucial in psoriasis [5]. It should be said that the differentiation of Th17 lymphocytes seems to be dependent on the action of prostaglandins, and studies in mice show that the EP2 and EP4 receptors are necessary for the differentiation of Th17 cells in psoriasis [114]. In addition, deletion of EP2 or EP4 in mice leads to a reduction of both inflammation and symptoms of psoriasis [114]. HETEs may also play a role in psoriasis development because their elevated levels, as well as higher expression of main enzymes responsible for HETEs generation, such as 12(R)-LOX [117,118], are observed in the affected skin. In the skin, HETEs may act as a chemoattractant for neutrophils and lymphocytes, leading to their accumulation [118,119]. Besides the skin, higher HETEs levels are observed in psoriatic peripheral blood lymphocytes [34]. In the case of these cells, HETEs cause a shift of Th into Th1 lymphocytes and decrease Th2 cytokines synthesis, so they enhance the differentiation of lymphocytes. Because the differentiation of lymphocytes into Th1 is linked with psoriasis in this way, HETEs may increase disease severity [120]. 

On the other hand, it is known that another group of lipid mediators formed in reactions catalyzed by LOXs and leukotrienes (LT) are able to regulate immune cells, but in allergic and not in autoimmune diseases [121].

As in psoriasis, there is also an increase in the activity of phospholipase A_2_ and COX-2 occurring in RA and SLE [122,123]. Consequently, in RA, higher levels of prostaglandins, especially PGD_2_ and PGE_2_, are observed in the synovial fluid, while in SLE, levels of PGD_2_ in the plasma are increased [124]. Nevertheless, the role of these prostaglandins is better known for RA. Namely, PGD_2_ is one of the factors responsible for enhanced proliferation and decreased apoptosis of chondrocytes [125]. On the other hand, PGE_2_ seems to be one of the most important pro-inflammatory factors in RA, and interactions between PGE_2_ and its receptors may be a key element in the pathogenesis of the disease. Studies in rats have shown that administration of an EP4 selective antagonist leads to inflammation and symptoms of RA [126]. Since antagonists of other EP receptors (EP1–EP3) do not influence the severity of RA, it seems that EP4 is the crucial one [126]. Moreover, deletion of the EP2 or EP4 receptors results in a reduction in disease severity in a mouse model of RA. This may further suggest that EP4 plays a key role in the pathophysiology of RA [78], and antagonists that inhibit signaling of the receptor lead to a reduction in inflammation [126]. In contrast, even residual EP2 signaling is sufficient to lead to the development of inflammation, since antagonists are unable to completely block signaling compared to genetic deletion of the receptor [78,126].

However, both prostaglandins and HETEs are involved in the pathophysiology of rheumatoid arthritis. Studies on genetically modified mice have shown that the elimination of 15-LOX, which impairs the synthesis of 15-HETE, leads to the alleviation of the symptoms of the disease. Conversely, administration of 15-HETE leads to a higher generation of TNF-α and IL-1 in the same model [127]. In humans, 15-LOX is also present in the synovial tissue of rheumatoid arthritis patients [128], suggesting that the 15-HETE generation plays a role in the pathophysiology of RA and that the results of studies in mice may be applicable to human.

#### 1.2.3. Non-Enzymatic Modifications

Another group of lipid mediators formed in ROS-dependent reactions consists of lipid peroxidation products. Depending on the mechanism of formation, these can be divided into reactive, electrophilic α, and β-unsaturated aldehydes, which are products of oxidative fragmentation and interact with compounds having nucleophilic centers such as lipids and proteins, or prostaglandin derivatives with lower chemical reactivity resulting from oxidative cyclization of phospholipid unsaturated fatty acids [129,130,131]. Aldehydes, mainly 4-hydroxynonenal (4-HNE) and malondialdehyde (MDA), can act as antigens for immune cells, leading to the production of antibodies [132]. It is known that 4-HNE enhances activation of TLR4, which may lead to activation of dendritic cells and therefore lymphocytes and other leukocytes [133]. Moreover, 4-HNE activates NF-κB, so it may therefore act as an activator of immune cells [134,135]. Since NF-κB is one of the most important transcription factors that regulate the production of pro-inflammatory cytokines, its activation leads to the onset of inflammation. 

Increased levels of 4-HNE and 4-HNE protein adducts have been observed in plasma and blood cells, as well as skin cells (keratinocytes and fibroblasts) of patients with psoriasis [33,136,137,138], causing higher expression of NF-kB and TNFα in blood cells [33]. This suggests that 4-HNE may be involved in NF-κB activation and inflammation in psoriasis. Similarly, higher levels of 4-HNE and 4-HNE protein adducts have been observed in RA and SLE [139,140,141]. Since reactive aldehydes and other lipid derivatives can act as autoantigens, antibodies to MDA and MDA–LDL adducts have been observed in the blood of patients with SLE [132]. However, antibodies to enzymatic lipid metabolism products, such as 9-HODE and 13-HODE, have also been observed in SLE, and their levels correlated with disease severity [132], while antibodies to MDA have also been observed in RA [142]. However, it is not known whether this increase in anti-lipid antibodies is significant for the development of SLE or RA, or whether it is merely a result of increased oxidative stress manifested by higher levels of oxidized molecules. Since immune complexes consisting of an antibody and its target are known to be powerful activators of the immune system, they are likely to be involved in the development or at least exacerbation of SLE. In psoriasis, autoantibodies to lipid derivatives have not yet been reported, although an increase in levels of these derivatives is observed [33]. Aldehydes form as a result of ROS-dependent fragmentation of polyunsaturated fatty acids and act as oxidative compounds that intensify oxidative stress. On the other hand, these molecules, particularly 4-HNE, are important pro-apoptotic agents that can enhance the pro-apoptotic pathways of immune cells and thus suppress inflammation [49].

Lipid cyclization products may also be involved in inflammatory reactions, and at least some of the effects of isoprostanes are due to their chemical similarity with prostaglandins; hence, isoprostanes may also act through prostaglandin receptors [129]. It has been shown that 8-isoPGE_2_ and 8-isoPGF_2α_ enhance the interactions between endothelial cells, macrophages, and neutrophils and thus increase migration of these cells to the site of inflammation [143,144]. Moreover, 8-isoPGF_2α_ can activate the MAPK pathway in macrophages causing higher production of IL-8 in the cells [145]. Because IL-8 is important in the differentiation of lymphocytes into Th1 cells, 8-isoPGF_2α_ may enhance inflammation in autoimmune diseases [91]. In contrast, isoprostanes can also act as anti-inflammatory agents by reacting with cysteine residues in IκB kinase (IKK). Typically, IKK phosphorylates NF-κB inhibitors, leading to their conjugation with ubiquitin and subsequent degradation and resulting in activation of NF-κB [146]. However, since 15-A2-isoprostanes interfere with this process, they lead to lower activation of NF-κB [146]. Higher levels of isoprostanes have been observed in most autoimmune diseases including psoriasis, SLE, RA, and in other conditions that are accompanied by oxidative stress [33,140,141]. Even though isoprostanes may play a role in modulation of immune cell function, they are so far mostly considered to be markers of oxidative stress, with their impact believed to be less important than the impact of other lipid derivatives in the case of immunity. 

This situation again shows two faces of ROS and induced by them the oxidative stress and its effects, in this case an increase in the level of isoprostanes. A similar response was generated by the effects of dimethylformamide (DMF)pharmacotherapy for multiple sclerosis [147], manifested by an increase in ROS production, which was considered to control the dysregulated autoimmune response of monocytes. Presumably, a similar situation applies to other autoimmune diseases, such as those that are the focus of this review. 

## 2. Conclusions

It can be assumed that oxidative stress that accompanies autoimmune diseases may intensify inflammation. In addition, there is ample evidence that reactive oxygen species (ROS) increases inflammation and activates immune cells. The products of ROS-dependent lipid metabolism are also generally considered as pro-inflammatory agents and are observed at higher levels in autoimmune diseases. The situation is more complicated for enzymatic lipid metabolism products as some of these may act as pro-inflammatory factors, while others as anti-inflammatory ones [112]. Furthermore, it appears that endocannabinoids can act in two directions depending on which receptor they activate; however, because CB2 receptors predominate in immune cells and most studies show that endocannabinoids reduce immune cell activity, increased endocannabinoid production in autoimmune diseases is usually considered as a compensatory mechanism that at least partially reduces inflammation. Perhaps the most perplexing situation is with eicosanoids, which are in part anti-inflammatory factors and increase differentiation of lymphocytes to Th2, while they are probably necessary for the development of both Th1 and Th2 responses. Despite this, complex interactions between lipids and the immune system are intensified during autoimmune diseases and, together with oxidative stress, act as important factors of their pathogenesis, which is still not entirely understood. 

## Data Availability

Data sharing not applicable.

## Abbreviations

2-AG	2-Arachidonylglycerol
4-HNE	4-Hydroxynonenal
4-ONE	4-Oxynonenal
AEA	Anandamide
CB	Cannabinoid receptor
COX	Cyclooxygenase
DAGL	Diacylglycerol lipase
DP	Prostaglandin D receptor
EP	Prostaglandin E receptor
IFNγ	Interferon γ
IL	Interleukin
IMQ	Imiquimod
IPP	Prostaglandin I receptor
LOX	Lipoxygenases
LTB4	Leukotriene B4
MDA	Malondialdehyde
NAPE-PLD	N-acylphosphatidylethanolamine phospholipase D
NAT	N-acyltransferase
NFκB	Nuclear factor kappa-light-chain-enhancer of activated B cells
Nrf2	Nuclear factor erythroid 2-like
OEA	Oleoylethanolamide
PGE	Prostaglandin D
PGE	Prostaglandin E
PGF	Prostaglandin F
PGI	Prostaglandin I
PGJ	Prostaglandin J
PLA_2_	Phospholipase A2
PLC	Phospholipase C
PUFAs	Polyunsaturated fatty acids
RA	Rheumatic arthritis
ROS	Reactive oxygen species
SLE	Systemic lupus erythematosus
Th	T helper lymphocytes
TLR	Toll like receptor
TNFα	Tumor necrosis factor α
TNFR	Tumor necrosis factor receptor
TRPV1	The transient receptor potential cation channel subfamily V member 1
TXA_2_	Thromboxane A2
